# Pedicle paradox: Duplicate vertebral pedicles creating a rare “pseudo foramen” with symptomatic foraminal stenosis

**DOI:** 10.1016/j.inpm.2025.100733

**Published:** 2026-01-08

**Authors:** Mark R. DeCotiis

**Affiliations:** Tri Rivers Musculoskeletal Centers, University of Pittsburgh Medical Center, 7500 Brooktree Road Suite 302, Wexford, PA, 15090, USA

**Keywords:** Pedicle duplication, Pseudo foramen, Radiculopathy, Neural arch, Hemivertebra

## Abstract

**Introduction:**

Duplication of a vertebral pedicle is a rare congenital anatomic variant with limited representation in the medical literature. Such anomalies are thought to arise during intrauterine or early postnatal phases of osseous development. Aberrant pedicle duplication can result in the creation of a “pseudo foramen,” with potential clinical implications that are not well characterized.

**Case:**

A patient presented with symptoms of painful burning sensations and paresthesias within the right groin and anterior thigh, consistent with upper lumbar radiculopathy. Advanced imaging of the lumbar spine revealed neuroforaminal stenosis associated with a “pseudo foramen” at L2, created by pedicle duplication. Together with clinical suspicion, results of a transforaminal epidural steroid injection supported the conclusion that the neuroforaminal stenosis at this level contributed to the patient's symptoms. This report examines the embryological origins and imaging findings of pedicle formation, considers the spectrum of pedicle duplication and semi-segmented hemivertebra, and reviews relevant imaging findings.

**Conclusion:**

This case highlights the importance of carefully reviewing lumbar imaging modalities. Not only is there observation of a rare duplication of vertebral pedicles, but also a thorough review of available literature suggests that this is the first case to specifically report on clinically significant stenosis occurring within a “pseudo foramen.”

## 1 Introduction

The vertebral pedicle forms part of the neural arch, providing an osseous bridge spanning the anterior vertebral body to the posterior lamina and lateral transverse process. Proper development of the pedicle is critical to the stability of the neural arch, and consequently, to the safety of the nervous system structures sheltered therein, including the spinal cord, nerve rootlets, and spinal nerves. Developmental abnormalities of the pedicles have previously been reported, including agenesis and hypoplasia [1]. Pedicles normally form bilaterally, but duplication of a single pedicle is a much rarer phenomenon.

Embryologically, early chondrification of the neural arch begins around week 6 of intrauterine life, enclosing the fledgling notochord and neural tube structures [2]. This yields to early ossification, around 9 weeks, which includes the development of the vertebral body and bilateral neural arches from respective primary ossification loci [2]. As the pedicles constitute elements of the neural arches, it is logical to conclude that pedicle duplication could result from an irregularity during primary ossification. Through childhood and adolescence, secondary ossification zones further develop features such as the transverse and spinous processes, fusing completely by the age of 25 years, in most cases [3]. Hypothetically, this timing could also yield irregularities in neural arch formation.

Neuroforamina of the vertebrae are typically bilaterally paired structures through which exiting spinal nerves traverse. Additionally, foramina can contain the sensory dorsal root ganglion, radicular and/or segmental medullary arteries, intervertebral veins, and lymphatic vessels pertaining to each spinal level. The foramina are bound anteriorly by the vertebral body, intervertebral disc, and posterior longitudinal ligament. Posteriorly, they are bound by the zygapophyseal joint complex, including the zygapophyseal capsule that conjoins and fuses medially with the ligamentum flavum. The medial border includes the dural sleeve that encases the exiting nerve rootlets as they merge into spinal roots. Superiorly and inferiorly, the foramina are bound by the inferior edge of the superior vertebral pedicle and the superior edge of the inferior vertebral pedicle, respectively. Importantly, deep adipose tissue provides cushioning to structures within the foramen. Myriad causes can lead to a reduction or thinning of epidural adipose within these regions, including direct causes such as foraminal disc extrusions or protrusions and zygapophyseal arthropathy or cyst formation, or indirectly, such as reduced intervertebral disc height contributing to narrowing between the superior and inferior pedicles. This can lead to imaging findings of neuroforaminal stenosis, which in turn, may generate radicular symptoms for the patient.

There are two reported cases of isolated pedicle duplication in the medical literature. Bhosle et al. observed a pedicle duplication at L4 in a 60-year-old female patient complaining of low back pain, but not radicular symptoms [4]. Their assessment indicated that each pedicle appeared thinner than the corresponding pedicles above and below the L4 level. As expected, the pedicle duplication formed a “pseudo foramen” between them, although the authors were uncertain if any spinal nerve traversed through it.

The other reported case of isolated pedicle duplication was observed coinciding with a thoracic compression fracture [5]. Although the case's 72-year-old female patient harbored medical comorbities related to osteoporosis such as chronic kidney disease and obesity, it was hypothesized that the presence of bilateral duplicate vertebral pedicles led to exaggerated segmental kyphosis, which created unequal axial loading to the thoracic vertebral body, eventually predisposing to fracturing.

Aside from isolated duplicate pedicles, there have been reports of other developmental anomalies of the osseous spine, such as hemivertebrae, which can exhibited duplicate pedicles in addition to other findings, duplicate laminae [6], duplicate cervical spinous processes [7], and even entire duplicate lumbar vertebral bodies [8,9]. To date, there have been no reported cases of duplicate pedicles, in any capacity, contributing to ipsilateral neuroforaminal stenosis with resultant radicular symptoms.

## 2 Case report

**Fig. 1:**
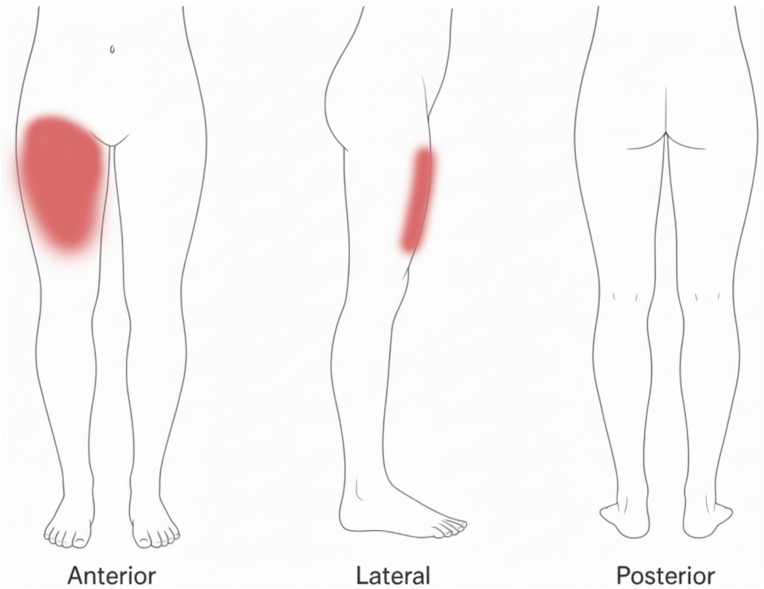
Patient's pain diagram.

A 53-year-old female presented with pain in the right groin and anterior thigh (Fig. 1). There were characteristic burning and paresthesia qualities to the pain, worse with weight-bearing, leaning forward, lifting, and walking. Her symptoms progressed to include pain with supination, making it challenging for the patient to sleep. X-rays of the pelvis and bilateral hips showed minimal degenerative changes of the right hip joint. A physical examination indicated no redness or erythema, mild discomfort with internal and external range of the motion of the hip, full strength, and mildly positive reproduction of concordant symptoms with flexion-adduction-internal rotation (FADIR) and flexion-abduction-external rotation (FABER) testing. The decision was made to proceed with a round of home exercises for the hip, coupled with prescriptions for meloxicam and gabapentin. After these modalities failed to significantly improve the patient's symptoms, and after discussion with the patient on the risks and benefits, a fluoroscopic-guided intraarticular hip corticosteroid injection was recommended for diagnostic and therapeutic purposes. Unfortunately, the injection provided only minimal relief in the short and medium term. A hip MRI arthrogram was also completed that showed no significant abnormalities within and around the right hip joint.

**Fig. 2:**
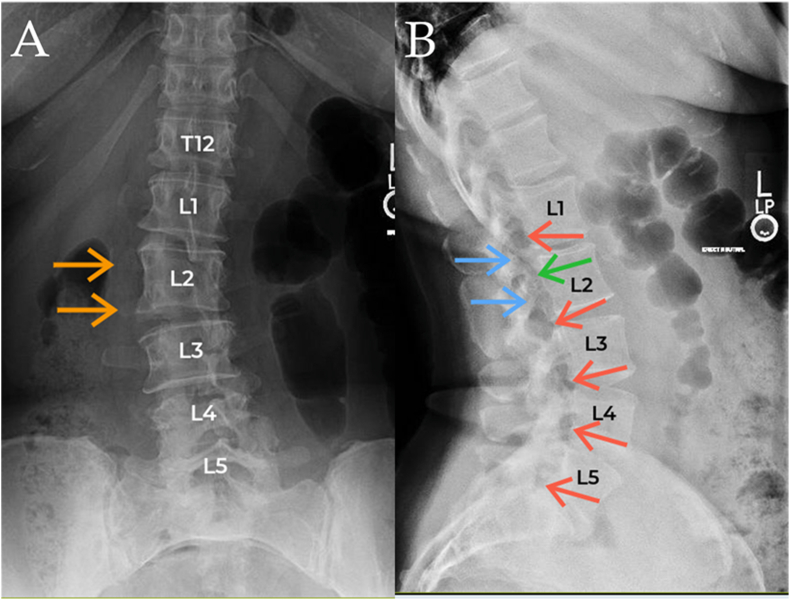
X-ray films of the thoracolumbar spine. The AP view (A) shows transitional anatomy as well as duplicate transverse process extending from the right L2 vertebral body (orange arrows). The lateral view (B) shows duplicate pedicles at the L2 vertebral body (blue arrows) as well as typically located intervertebral foramina (red arrows) and “pseudo foramen” located between the duplicate pedicles (green arrow). Note the “pseudo foramen's” slightly posterior location compared to other typical foramina. (For interpretation of the references to colour in this figure legend, the reader is referred to the Web version of this article.)

**Fig. 3:**
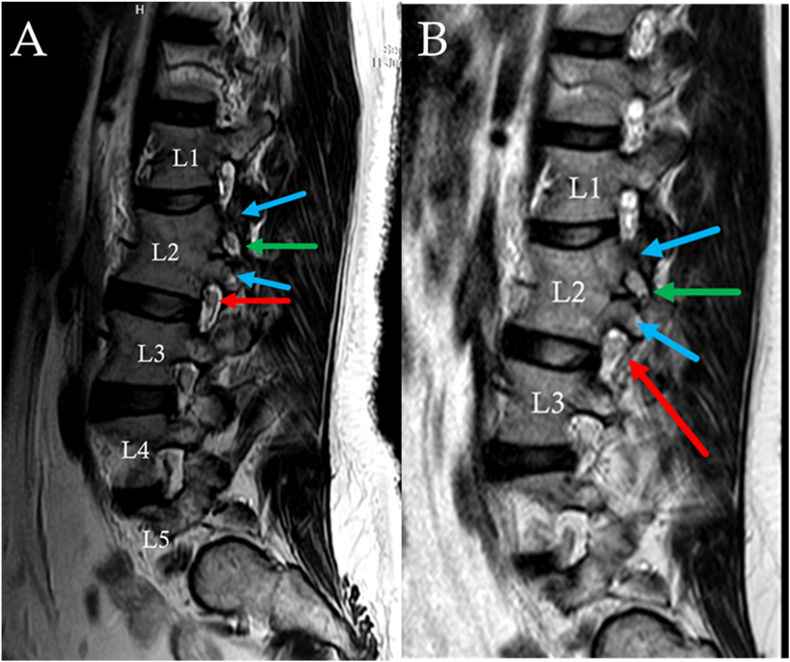
T2-weight MRI images of the lumbar spine. Right-sided sagittal neuroforaminal cuts shown of the patient's 2025 (A) and 2022 (B) studies. Both show duplicate pedicles (blue arrows), typical foramen (red arrow), and “pseudo foramen” (green arrow) containing an exiting nerve root with surrounding moderate neuroforaminal stenosis. (For interpretation of the references to colour in this figure legend, the reader is referred to the Web version of this article.)

**Fig. 4:**
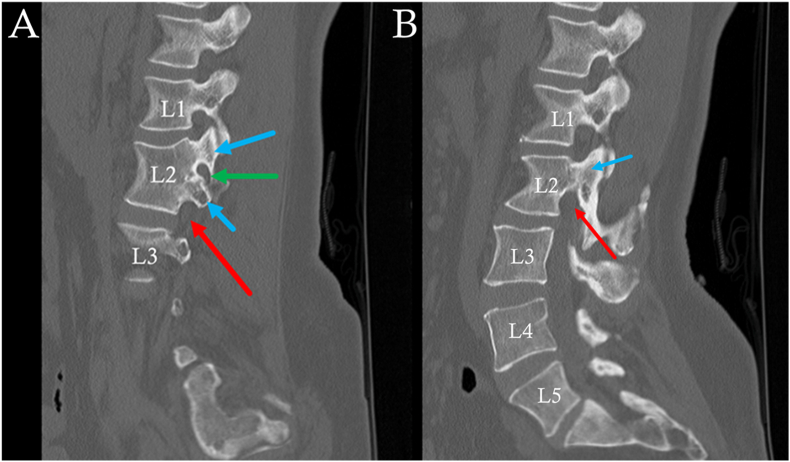
Sagittal CT Bone images of right (A) and left (B) lumbar spine. Appreciate multiple pedicles (blue arrows) on the right, and single pedicle on the left. Corresponding “pseudo foramen” (green arrow) compared to typical foramen (red arrows). (For interpretation of the references to colour in this figure legend, the reader is referred to the Web version of this article.)

**Fig. 5:**
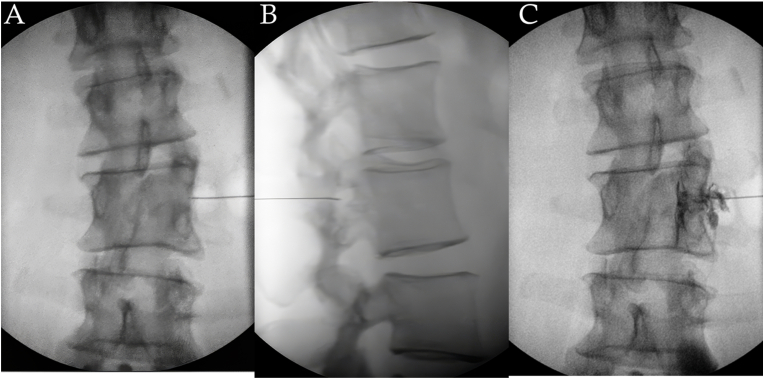
Fluoroscopic images from the patient's transforaminal epidural steroid injection. The “pseudo foramen” was targeted with corresponding AP (A) and lateral (B) imaging indicating appropriate needle placement prior to contrast flow medial to the pedicle (C).

Gradually, the patient also began complaining of very mild upper and mid lumbar discomfort, which had not been her primary area of discomfort at the time of previous evaluations. Physical examination indicated mild discomfort with flexion and rotation, with intact strength and sensation throughout the lower extremities. There was slight discomfort within the anterior groin with prone knee bend/reverse slump test. AP and lateral lumbar X-ray films (Fig. 2) indicated lumbosacral transitional anatomy, with the identification of a sacralized L5 vertebral body and presumed hypoplastic 12th ribs. With this radiological numbering in mind, there was observed a misshapen L2 vertebral body as well as a duplicate pedicle, transverse process, pseudo facet joint, and resulting neuroforamen on the right, at this same L2 level. Subsequently, a lumbar MRI was completed, which showed moderate neuroforaminal stenosis at this “pseudo foramen” on the symptomatic right side (Fig. 3A). It was found that the patient had a lumbar MRI completed in 2022, with evidence at that time as well of the “pseudo foramen” and similar stenosis (Fig. 3B). CT scan further elucidated the bony abnormality (Fig. 4). With failure of conservative modalities, interventional options were discussed to help manage the patient's symptoms stemming from right L2 radiculopathy. Initially, an interlaminar epidural steroid injection was completed at the level of L2-3 with observable superior epidural contrast flow. This failed to significantly improve the patient's symptoms. Next, a transforaminal epidural steroid injection was completed at the “pseudo foramen” (Fig. 5). The patient noted reproduction of her concordant symptoms during placement of the injectate: 1 % Lidocaine in combination with dexamethasone 15 mg. The patient was given a pain diary to monitor her symptoms.

Her pain diary showed 100 % improvement of her concordant groin/thigh symptoms for 4 days. This was followed by gradual return of pain, burning, and paresthesias in her known distribution. She has sought consultation and is contemplating surgical options.

## 3 Discussion

The embryological development of the patient's duplicate pedicles does not neatly fit into a case of isolated pedicle duplication nor a case of hemivertebrae, but incorporates aspects of each. Isolated pedicle duplication includes doubling of ipsilateral or bilateral pedicles and possibly posterior elements. It rarely leads to focal scoliosis, and vertebral body shape tends to be normal. Hemivertebrae, including the semi-segmented subtype that this case resembles, develop due to failure of one of the lateral, primary chondrification centers of the vertebral body [10]. This can lead to duplication of posterior elements and wedge or trapezoidal-shaped vertebral bodies, focally altering spinal biomechanics that contribute to kyphosis and scoliosis [11]. Hemivertebrae can also be associated with more systemic syndromes, such as Pfeiffer, Crouzon, and Kabuki syndromes [10,12]. The case presented includes an abnormally shaped vertebral body with focal scoliosis, although the patient did not suffer from associated systemic syndromes. Therefore, it is likely the patient's anatomy lies somewhere on a spectrum of embryological spinal deformity between these entities.

The salient and unique feature of this case report is the primary “pseudo foraminal” stenosis caused at the level of duplicate pedicles, on the convex side of the patient's focal scoliosis. Several cases have detailed the direct and indirect clinical implications of hemivertebrae, but none describe the novelty presented in this case. Published case reports indicate examples of biomechanical changes contributing to nearby disc protrusions resulting in radiculopathy with butterfly vertebrae, a close embryological relative to hemivertebrae [10,13,14], a direct protrusion of a S1 butterfly vertebrae body causing lateral recess stenosis [15], and an instance of posterior hemivertebrae causing severe central stenosis [16]. As far as cases of hemivertebrae with duplicate pedicles, Nagashima et al. reported foraminal stenosis on the concave/contralateral side to the duplication, likely caused by degenerative changes [17]. In another case, duplicate pedicles contributing to focal scoliosis causing concave/contralateral “far out” radiculopathy of the L5 nerve root on the sacral ala [18]. Interestingly, this case also observed a “bony tunnel” between the duplicate pedicles on the convex side, through which there was an exiting nerve root, but there was no symptomatic stenosis [18]. Regardless of the nomenclature used to categorize the osseous abnormality in this case, review of the available literature suggests that this is the first published report specifically observing clinically significant stenosis across a “pseudo foramen” created by duplicate pedicles. The clinically relevant stenosis occurs on the convex side of the possible hemivertebra, which has also not been reported in the literature.

Review of the patient's sagittal MRI imaging indicates an expected and appropriate 1:1 association of spinal nerves leaving segmental neuroforamina throughout the lumbar spine (Fig. 3). It can be speculated that at least some components of the patient's upper lumbar (i.e. L1, L2, and L3) spinal nerves are traversing through this stenotic “pseudo foramen.” It would be tedious and challenging to fully characterize which nerve rootlets are contributing to this affected spinal nerve. We must recall her transitional anatomy. Full-spine imaging was not obtained. Nonetheless, if we try to affix the patient's symptoms to our known dermatomal patterning, one hypothesis includes nerve rootlets from L1 and L2 were merged upon the medial aspect of the “pseudo foramen” to form the impacted “pseudo spinal nerve.” Alternatively, rootlets intended to form L2 may have divided through both the superior/“pseudo foramen” and the inferiorly displaced foramen that exhibits minimal neuroforaminal stenosis. Neither of these hypotheses could be adequately tested with the available axial imaging from the patient's MRI, but perhaps additional imaging could contribute here. However, Chan et al. reported on a conjoined L4-5 nerve root at the level of a fully segmented hemivertebra that was not clearly seen on pre-surgical MRI, and was only adequately visualized once decompressive surgery had commenced [19]. As the presented scenario shows some similarities to a hemivertebral anomaly, perhaps it is possible for a conjoined L1-2 nerve root in this case.

The case study has several limitations. The patient's transitional anatomy could be further elucidated with full spine imaging. This would help clarify the presence of hypoplastic ribs as they pertain to numbering of the distal lumbar and lumbosacral vertebral bodies. Thus, providing more accurate numbering of the spinal nerves that are impacted by the stenosis. Secondly, a selective nerve root block would provide more specific support for the clinical conclusion of a single level radiculopathy. Although more selective than an interlaminar epidural steroid injection, the contrast flow pattern from the transforaminal epidural steroid injection completed in this case did show some superior and inferior migration (Fig. 5).

In summary, upper lumbar radiculopathy should be included in a differential diagnosis of groin and thigh discomfort. This case underscores the value of meticulous clinical evaluation and thorough review of imaging modalities that led to the identification of not only rare aberrant anatomy but also distinctive symptomatology, previously undescribed in medical literature.

## Informed consent

Informed consent was obtained from the patient referred to in the case. She understands that her personal details and identity would not be included, but only her age, symptoms, medical course, and imaging findings would be reported on.

## Declaration of competing interest

The author declares that he has no competing interests, financial or personal, that would have influenced the content of this report.
